# Self‐Assembling Anti‐Freezing Lamellar Nanostructures in Subzero Temperatures

**DOI:** 10.1002/advs.202309020

**Published:** 2024-02-17

**Authors:** Hongyao Yin, Weiluo Guo, Runxi Wang, James Doutch, Peixun Li, Qiang Tian, Zhuo Zheng, Lingzhi Xie, Yujun Feng

**Affiliations:** ^1^ State Key Laboratory of Polymer Materials Engineering Polymer Research Institute Sichuan University Chengdu 610065 P. R. China; ^2^ Institute of New Energy and Low‐Carbon Technology Sichuan University Chengdu 610065 P. R. China; ^3^ ISIS Neutron and Muon Source, Science and Technology Facilities Council, Rutherford Appleton Laboratory Harwell Campus OXON Didcot OX11 0QX UK; ^4^ State Key laboratory of Environment‐Friendly Energy Materials, School of Materials and Chemistry Southwest University of Science and Technology Mianyang 621010 P. R. China

**Keywords:** anti‐freezing, lamellar nanostructure, lyotropic liquid crystal, subzero temperature, supramolecular self‐assembly

## Abstract

The requirement for cryogenic supramolecular self‐assembly of amphiphiles in subzero environments is a challenging topic. Here, the self‐assembly of lamellar lyotropic liquid crystals (LLCs) are presented to a subzero temperature of −70 °C. These lamellar nanostructures are assembled from specifically tailored ultra‐long‐chain surfactant stearyl diethanolamine (SDA) in water/glycerol binary solvent. As the temperature falls below zero, LLCs with a liquid‐crystalline L_α_ phase, a tilted L_β_ phase, and a new folded configuration are obtained consecutively. A comprehensive experimental and computational study is performed to uncover the precise microstructure and formation mechanism. Both the ultra‐long alkyl chain and head group of SDA play a crucial role in the formation of lamellar nanostructures. SDA head group is prone to forming hydrogen bonds with water, rather than glycerol. Glycerol cannot penetrate the lipid layer, which mixes with water arranging outside of the lipid bilayer, providing an ideal anti‐freezing environment for SDA self‐assembly. Based on these nanostructures and the ultra‐low freezing point of the system, a series of novel cryogenic materials are created with potential applications in extremely cold environments. These findings would contribute to enriching the theory and research methodology of supramolecular self‐assembly in extreme conditions and to developing novel anti‐freezing materials.

## Graphical abstract

Anti‐freezing lamellar lyotropic liquid crystals with temperature tolerance to −70 °C are realized through a supramolecular self‐assembly of an amphiphile in water/glycerol binary solvent. As temperature falls below 0 °C, crystals with L_α_ phase, tilted L_β_ phase, and a new configuration are obtained consecutively. The results enrich the understanding of supramolecular self‐assembly in extreme environment, and may be useful to create novel materials.

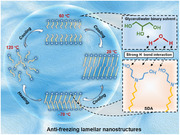

## 1 Introduction

Molecular self‐assembly is important for understanding matter present in nature and for creating new materials; carrying it out in extreme conditions will further our understanding.^[^ ^1^ ^]^ Amphiphilic molecules continuously attract attention from scientific and industrial communities because their micro‐ and nano‐sized self‐assemblies are widely used to mimic biological structures, and to create various novel materials.^[^ ^2^ ^]^ Lamellar nanostructures (i.e., lamellar lyotropic liquid crystals, LLCs) are among the typical assembled architectures with alternating layered surfactant molecules. This type of structure has been extensively used for complex fluid,^[^ ^3^ ^]^ drug delivery,^[^ ^4^ ^]^ model membranes,^[^ ^5^ ^]^ and soft templates for the synthesis of nano‐materials,^[^ ^6^ ^]^ and it has received increasing attention in recent decades.^[^ ^7^ ^]^ Although lamellar structure is a relatively well characterized subject, relating to the fabrication of the lamellar phase, it has not yet been explored at subzero temperatures. This limitation impedes the deep understanding and development of self‐assembly theory; it also limits the assembled structures’ applications in extremely cold environments, such as the polar regions or outer space. For example, it cannot currently mimic the functionality of the cell membrane or synthesize mesoporous materials using amphiphiles assemblies in a frozen environment. The key challenges come from the high freezing point of an aqueous system, the poor solubility of conventional amphiphiles at low temperatures, and the uncertainty of self‐assembly behavior in extreme environments. In consideration of the current demands of various anti‐freezing materials, the development of cryogenic self‐assembly of lamellar nanostructures has assumed a degree of urgency.

Herein, we address these unmet challenges and report the first successful attempt of assembling lamellar nanostructures including lamellar sheets and liquid crystals down to −70 °C. Inspired by the ability of plants and animals to survive in extremely cold weather through preventing generation of ice crystals in their cells, researchers have found and used many chemical cryoprotectants, including inorganic salts, organic solvents, and polymers, to lower water's freezing point.^[^ ^1^, ^8^ ^]^ Glycerol is an ideal freezing point inhibitor that can create cryogenic environment for amphiphile assembly owing to its non‐toxicity, relatively high Gardon parameter, and good miscibility with water.^[^ ^9^ ^]^ The freezing temperature of water/glycerol mixture can be simply regulated via changing the ratio between the two components, and the lowest value reaches −46.5 °C when the glycerol content is 66.5 wt%.^[^ ^10^ ^]^ Nevertheless, higher alcohol content weakens the aggregating capability of amphiphiles.^[^ ^11^ ^]^ Furthermore, both solvo‐phobic interaction and hydrogen bonds (H‐bonds) significantly contribute to forming stable assembled architectures.^[^ ^12^ ^]^ With this in mind, we propose a strategy of using a surfactant bearing a long hydrophobic tail and a large head group with multiple H‐bond donors and/or acceptors as the amphiphilic candidate for self‐assembly in water/glycerol medium to realize anti‐freezing lamellar assemblies in subzero temperatures.

**Scheme 1:**
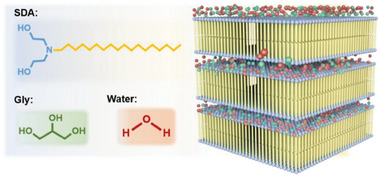
Chemical structure of SDA, glycerol, and water molecules, with the schematic representation of self‐assembled anti‐freezing lamellar LLCs.

As such, we selected 2,2′‐(octadecylazanediyl)diethanol (SDA, **Scheme** **1**) specifically tailored with a C_18_‐tailed chain, and a head group bearing three active points for generating H‐bonds to self‐assemble in water/glycerol mixture down to subzero temperatures. Extensive experimental studies demonstrated that SDA had a very strong ability to form lamellar nanostructures, generating lamellar sheets at 0.1 wt% and LLCs at 0.5 wt%. Molecular simulation studies revealed that both the ultra‐long alkyl chain and head group of SDA played a crucial role in the formation of lamellar nanostructures. Glycerol cannot penetrate the lipid layer, which mixed with water arranging outside of the lipid bilayer, providing an ideal anti‐freezing environment for SDA self‐assembly. Upon decreasing the temperature, the disordered L_α_ liquid‐crystalline phase transformed into the tilted L_β_ gel phase at ≈40 °C, which was further transformed into a new folded configuration while cooling down to below 0 °C. By optimizing this system, we could create LLCs with the lowest temperature tolerance of −70 °C. Within these cryogenic nanostructures, various anti‐freezing materials with unique rheological properties were created, with potential applications in extreme environments.

## 2 Results and Discussion

### 2.1 Fabrication of Cryogenic Self‐Assembly System

**Figure 1:**
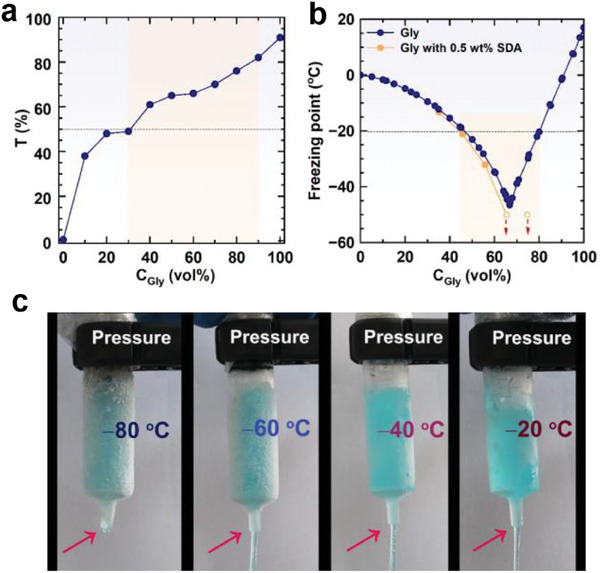
a) Light transmittance at 550 nm of the 0.5 wt% SDA solution with various glycerol content at 20 °C. b) Freezing point of water/glycerol co‐solvent (data are replotted from the literature ref.[10]) and 0.5 wt% SDA solution with various glycerol content. c) Fluidity of 1.0 wt% SDA fluid with 70 vol% glycerol at different temperatures.

Keeping in mind that the low freezing point of self‐assembly mediums and good solubility at subzero temperatures are the prerequisites of cryogenic self‐assembly, we commenced this study with an investigation of SDA solubility in water/glycerol mixture and its freezing temperature. We found that SDA did not dissolve in pure water or pure glycerol, but rather in their mixture. When 0.5 wt% SDA was dissolved in pure water, only a turbid mixture was obtained (Figure S2, Supporting Information). The mixture then gradually became transparent when glycerol was added. Light transmittance at 550 nm of the mixture was smaller than 50% when glycerol content was less than 30 vol%, but it sharply increased to over 60% in the presence of 40 vol% glycerol (**Figure** **1**a), indicating that the SDA was well dissolved in this case. The transmittance continuously rose with the increase in glycerol content and reached 91% for pure glycerol. However, a large number of solid particles adhered to the bottle wall or floated atop the mixture in the sample with pure glycerol. Based on the results of light transmission and visual observation, we determined that SDA could only dissolve in the binary solvent containing 40−90 vol% glycerol, so we therefore focused on this range.

The freezing point of water/glycerol binary solvent was investigated by Lane in 1925.^[^ ^10^ ^]^ As shown in the blue line in Figure 1b, with the increase in glycerol content, the freezing point decreased to the minimum value of −46.5 °C at 66.5 wt% glycerol, followed by an increase. We then investigated the freezing point of co‐solvent containing 0.5 wt% SDA, as presented in the yellow line in Figure 1b. The addition of 0.5 wt% SDA led to a lower freezing point; in particular, the samples containing 65.4 and 74.6 wt% glycerol showed a value even lower than −50 °C beyond the accessible range of the instrument. The decline in freezing point was presumably caused by the destruction of H‐bonds between solvent molecules and the formation of assembled microstructures.^[^ ^13^ ^]^ Fluidity results show that 1.0 wt% SDA solution with 50 vol% glycerol could flow well at −20 °C (Figure S3 and Movie S1, Supporting Information), while the fluid with 70 and 90 vol% glycerol could flow at −60 °C (Figure 1c; Figure S4, Movies S2 and S3, Supporting Information), which is in line with the freezing point results. In short, this binary solvent provides an ideal anti‐freezing medium to study the SDA self‐assembly behavior at subzero temperatures.

### 2.2 Microstructure of Self‐Assemblies

**Figure 2:**
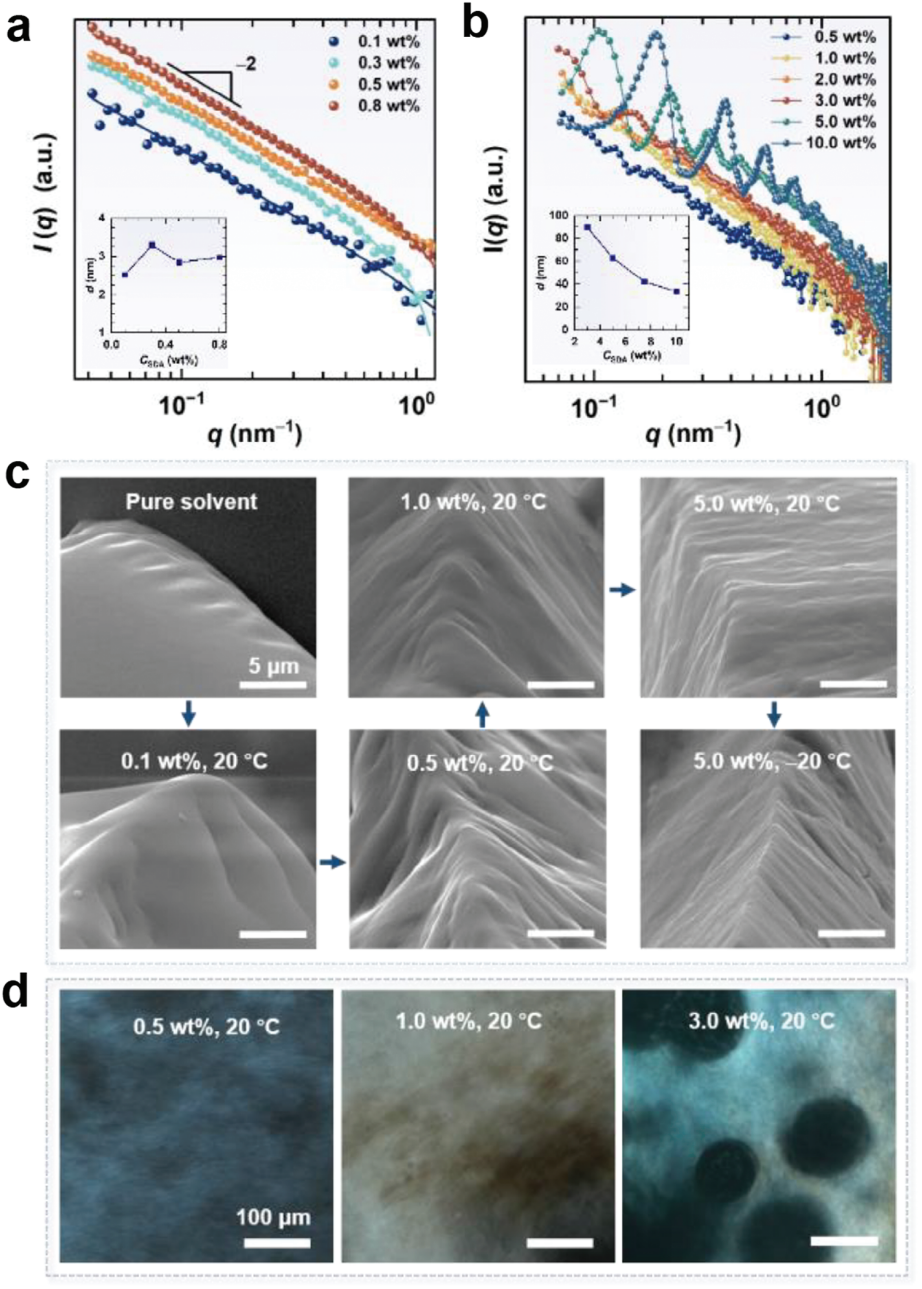
Microstructure of SDA self‐assemblies in water/glycerol binary solvent with 50 vol% glycerol. a) SANS and b) SAXS profiles at 20 °C, c) Cryo‐SEM images, and d) POM images. The inset in (a) and (b) is the bilayer thickness and the interlayer distance as a function of SDA concentration, respectively. The scale bar is 5 µm for cryo‐SEM images and 100 µm for POM images.

Exhibited in **Figure** **2**a are the small**‐**angle neutron scattering (SANS) curves of samples containing 0.1−0.8 wt% SDA in the binary solvent with 50 vol% glycerol. All these curves showed a *q* ^−2^ power law decay in the low‐*q* region, suggesting the presence of lamellar structures.^[^ ^14^ ^]^ The intensity increased with SDA concentration, reflecting the increase in the number density of lamellar structures. Analytical modeling of the whole SANS profile was achieved to a high goodness‐of‐fit using a lamellar model in SasView software. Fitting results show that the lipid bilayer thickness was ≈3 nm, as shown in the inset of Figure 2a.

Samples with higher SDA concentrations were characterized by small‐angle X‐ray scattering (SAXS). As exhibited in Figure 2b, Bragg peaks with the relative position of 1:2:3 were observed when SDA concentration reached 3.0 wt%, strongly supporting the formation of LLCs.^[^ ^15^ ^]^ As SDA concentration increased, the peaks became profound and shifted to higher *q* values. The interlayer spacing (*d*), calculated by the Bragg equation,^[^ ^16^ ^]^ was 89.7 nm for 3.0 wt% SDA, and decreased to 33.6 nm as the SDA concentration increased to 10.0 wt% (Figure 2b, inset). In addition, Bragg peaks become sharper with the increasing SDA concentration, suggesting the presence of periodic lamellar structure with improved regularity. From the calculated lipid bilayer thickness and the interlayer spacing, it can be derived that the solvent thickness between the adjacent lipid layer was ≈60 nm when the SDA concentration was 5.0 wt%. This solvent thickness is several times larger than that in the conventional lamellar phase.^[^ ^15^, ^17^ ^]^ It may be attributed to the superior capability of SDA in forming lamellar nanostructures.

Cryogenic scanning electron microscopy (cryo‐SEM) and polarizing optical microscopy (POM) observations were then used to directly examine the assembled morphology. Smooth surfaces without assembled aggregates were visible in the cryo‐SEM image for the pure co‐solvent, while wrinkled morphology with smooth edges appeared when 0.1 wt% SDA was added (Figure 2c), suggesting the formation of aggregates. However, the POM image for this sample displayed only a dark background (Figure S5, Supporting Information), indicative of an isotropic phase. Combining these results with the SANS data fitting, we inferred that lamellar sheets were formed but did not stack in long‐range order. When the SDA concentration increased to 0.5 wt%, a large area of lamellar layers was observed in the cryo‐SEM image and an oily streak texture was seen in the POM image (Figure 2d), which confirms the formation of LLCs,^[^ ^18^ ^]^ though no evident Bragg peaks were found from the SAXS measurement (Figure 2b). As the SDA concentration further increased, the edge of the lamellae became clearer and sharper, the order of the lamellar layers was improved, and the lamellar stacks became closer, which agrees with the SAXS results. Such morphology variations with SDA concentration reflect the significant increase in the lamellar phase size and the stacking order.

From the above analysis, we know that SDA has a strong capability to form lamellar assemblies in water/glycerol co‐solvent. It formed lamellar sheets at concentrations as low as 0.1 wt%, and these sheets grew and transformed into LLCs when the concentration reached only 0.5 wt%. Normally, much higher surfactant concentrations (i.e., >10 wt%) were required for conventional systems to form LLCs in previous studies.^[^ ^15^, ^17^, ^19^ ^]^ Comparisons with the literature suggest that this is the lowest surfactant concentration to form LLCs reported to date.

**Figure 3:**
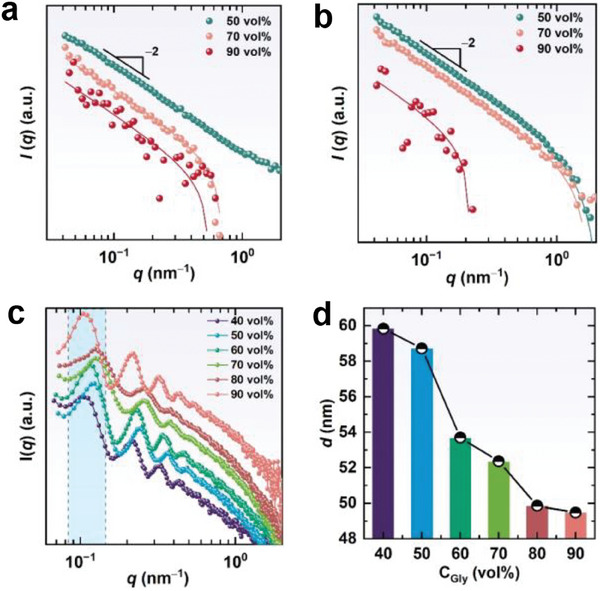
SANS profiles of a) 0.5 wt% and b) 0.8 wt% SDA, and c) SAXS profiles of 5.0 wt% SDA in a water/glycerol binary solvent with various glycerol contents at 20 °C. d) The interlayer distance as function of glycerol concentration.

The solvent effect on the microstructure was then investigated. As depicted in **Figure** **3**a,b, the increase in glycerol content led to an obvious decrease in the neutron scattering intensity, but the scattering curves maintained the *q* ^−2^ power law behavior in a low‐*q* region. Fitting results indicate that the bilayer thickness almost doubled when glycerol content rose from 50 to 90 vol%. Oily streak textures were also observed in the POM images for the samples with 70 and 90 vol% glycerol (Figure S5, Supporting Information), suggesting the presence of LLCs. SAXS characterization of the 5.0 wt% SDA solution showed that Bragg peaks gradually decayed and shifted to a higher *q* value as the glycerol content increased (Figure 3c), reflecting the decrease in the interlayer spacing and the decrease in the regularity. As shown in Figure 3d, the d‐spacing declined from 59.8 to 49.5 nm when glycerol content increased from 40 to 90 vol%. Combining the SANS and SAXS results, we posit that the increase in glycerol content does not destroy the lamellar phase, but results in the swelling of the lipid bilayers.

### 2.3 Lamellar Nanostructures at Subzero Temperatures

**Figure 4:**
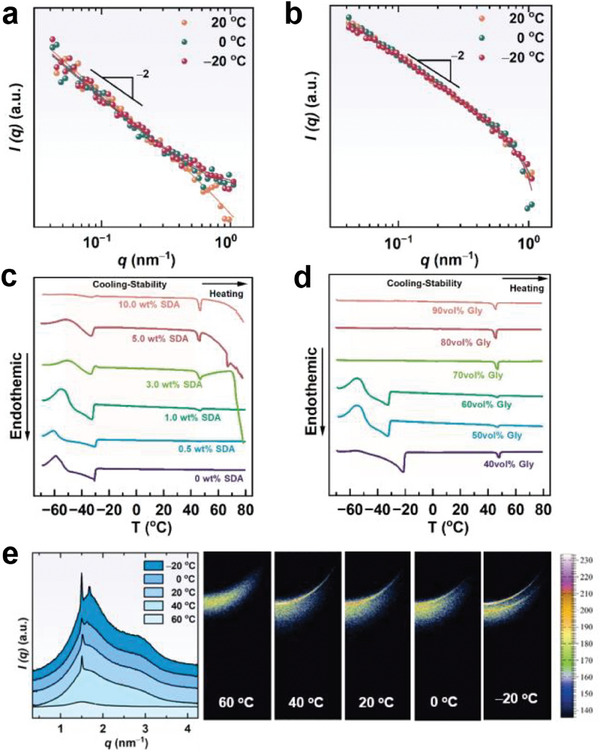
SANS profiles of a) 0.1 wt% and b) 0.3 wt% SDA fluid with 50 vol% glycerol. DSC thermograms obtained for c) SDA fluid at different concentrations with 50 vol% glycerol and d) 1.0 wt% SDA fluid with various glycerol contents. e) 1D WAXS profiles and 2D WAXS images of 5.0 wt% SDA fluid with 50 vol% glycerol.

The good solubility and excellent self‐assembly capability of SDA in water/glycerol binary solvent provide an opportunity to investigate self‐assembly behavior at subzero temperatures. Figure 4a,b shows the SANS profiles of 0.1 and 0.3 wt% SDA solutions from 20 to −20 °C, and Figure S6 (Supporting Information) displays the SANS profiles of 0.5 and 0.8 wt% SDA solutions from 60 to −20 °C. All of the profiles show the same *q* ^−2^ behaviors in the low‐*q* zone demonstrating that lamellar structures remained stable as temperature decreased to below 0 °C. Interestingly, cryo‐SEM observation showed that a temperature decrease from 20 to −20 °C significantly improved the regularity of the lamellar layers stacking (Figure 2c).

To gain further insights into the aggregate microstructure at temperatures far below 0 °C, we turned to DSC technique. Figure 4c shows the DSC curves of fluids containing 50% glycerol with various SDA concentrations. Two thermal transitions, one exothermic and one endothermic, were observed for the pure binary solvent upon heating from −70 to 80 °C. The exothermic transition with the peak at −58.75 °C can be attributed to the cold‐crystallization of super‐cooled water.^[^ ^20^ ^]^ This phenomenon was only observed within a narrow glycerol content range for this binary solvent, and it indicates the presence of an intermediate water phase in the mixture. The subsequent endothermic event with a peak at −30.89 °C was due to ice melting. The addition of SDA forming lamellar structures in the co‐solvent did not cause a new thermal event from the freezing point to ≈40 °C, suggesting that there was no structure transition of the assemblies in this temperature range. Namely, lamellar phases are always present in subzero temperatures.

Upon further increasing the temperature, we observed that a new endothermic peak appeared at ≈46 °C, which became more obvious with increasing SDA concentration. SANS curves with *q* ^–2^ behavior in the low‐*q* region at 40 and 60 °C (Figure S6, Supporting Information) for 0.5 and 0.8 wt% SDA solutions confirm the presence of lamellar nanostructures. Thus, this new endothermic peak might be the result of a phase transition in lamellar assemblies. Wide‐angle X‐ray scattering (WAXS) was performed from 60 to −20 °C for the 5.0 wt% SDA solution to verify our conjecture. At 60 °C, no peak was observed, suggesting that the assemblies were in a liquid‐like lamellar phase state (i.e., a lamellar liquid‐crystalline (L_α_) phase) in which the alkyl groups were disordered. While cooling down to 40 °C, a sharp crystalline peak appeared at *q* = 1.5 nm^−1^, corresponding to hydrocarbon chains with an inter‐acyl chain distance of 4.2 nm, indicating that the assemblies were in the gel (L_β_) phase state in which the alkyl chains that made up the bilayer were packed in an ordered state. After further cooling down to 0 °C, a second peak at *q* = 1.6 nm^−1^, corresponding to a shorter chain‐to‐chain distance of 3.9 nm, was observed simultaneously, and this scattering intensity was enhanced as the temperature decreased to −20 °C. The reason for this is that the temperature decrease results in the crystallization of SDA alkyl chains in the L_α_ phase, thus transforming into the L_β_ phase. Subzero temperatures benefit the formation of more stable packing of alkyl chains, leading to a second transition at lower temperatures. This novel example of alkyl chain configuration transition in the lamellar phase should be ascribed to the binary solvent that provides an anti‐freezing self‐assembly environment at subzero temperatures.

Varying glycerol contents led to different thermal events (Figure 4d). The sample containing 40 vol% glycerol only showed an endothermic peak at −21.64 °C. When glycerol increased to 60 vol% or a higher value, both the cold‐crystallization exothermic and ice‐melting endothermic peaks disappeared. However, the endothermic peak at ≈46 °C remained stable as glycerol content changed, revealing that the L_α_−L_β_ transition occurred with a temperature decrease, regardless of solvent composition. These results indicate that lamellar structures are present in subzero temperatures of at least −70 °C. Our work serves as a novel example of the lamellar phase being fabricated in a subzero environment.

It is worth nothing that the freezing point of the binary solvent gradually disappeared upon increasing SDA (Figure 4c) or glycerol (Figure 4d) concentration within the accessible measurement range. This intriguing phenomenon can be attributed to the variations in assembled nanostructures. The increase in SDA concentration, on one hand, leads to formation of a greater number of H‐bonds with water, while on the other hand, it renders a sharp decrease of the interlayer distance (Figure 2b). The dense alternating lamellar layers act as a network that separates and confines water molecules, preventing them from freezing.^[^ ^13^ ^]^ Similarly, an increase in glycerol concentration causes swelling of the lipid bilayer and consequently decreases interlayer distance (Figure 3d), thereby enhancing confinement of water molecules. Therefore, both glycerol introduction and dense LLCs formation contribute significantly to the dramatical reduction in the freezing point.

### 2.4 Mechanism of Lamellar Nanostructure Formation

**Figure 5:**
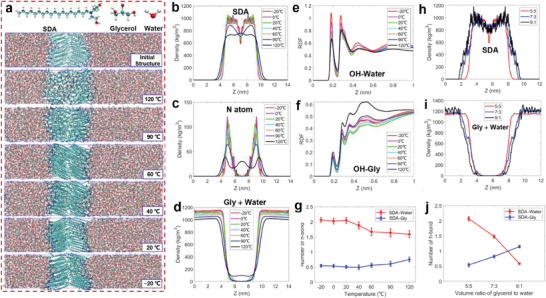
a) Simulation snapshots of SDA in water/glycerol binary solvent with 50 vol% glycerol. Density distribution of b) SDA, c) nitrogen atoms, and d) “water + glycerol” at different temperatures. RDF between e) hydroxyl of SDA and water, and f) hydroxyl of SDA and glycerol. g) The variation of H‐bonds number with temperature. Density distribution of h) SDA, i) “water + glycerol,” and j) the variation of H‐bonds number in co‐solvent with different glycerol/water ratios at −20 °C.

To gain insights into the mechanism on the molecular level of forming such lamellar nanostructures, we carried out molecular dynamics (MD) simulations from −20 to 120 °C. The schematic of the MD simulation system is shown in Figure S1 (Supporting Information). An initial rough lamellar configuration was constructed using 100 SDA molecules (50 pairs) in binary solvent with 50 vol% glycerol. After running 200 ns at 120 °C, a disordered aggregate was obtained, where SDA molecules were arranged out of order in a solvo‐phobic region (**Figure** **5**a). As the temperature decreased to 90 °C and 60 °C, the L_α_ phase and the conventional tilted L_β_ phase were respectively formed. When the temperature further decreased to below 20 °C, the SDA alkyl chains were packed in a new folded configuration with an angle of ≈140°. This configuration exactly agrees with the shortening of the inter‐acyl chain distance observed from the WAXS data, as the temperature decreases to below 0 °C.

Depicted in Figure 5b is the SDA density distribution, which had a symmetric distribution, indicating that the SDA molecules were arranged to form a regular bilayer on both sides. The distribution curves almost overlapped at −20 and 0 °C, and then the peak density slightly decreased from 20 °C. The fluctuation of the curves is due to the molecular structure and lower thermal motion. As the temperature increased, the curve became smoother, which also indicates that the carbon chains of SDA can move more freely. As the density peak decreased from 60 °C, the sharp peaks started to become smooth and broad, demonstrating that SDA can bend or move and that the bilayer gradually starts to break down. Until 120 °C, the peak of density distribution was absent, which means that all SDA molecules were randomly and uniformly arranged in the solvo‐phobic area. It can also be seen from the N atom density distribution of SDA in Figure 5c that the bilayer was completely destroyed at 120 °C, and this trend is consistent with the density distribution of SDA. The variation of the SDA alkyl chain length with temperature (Figure S7, Supporting Information) shows that the hydrophobic length stabilized at 2.05 nm from −20 to 60 °C and then dramatically decreased to 1.65 nm as the temperature increased to 120 °C. These results clearly demonstrate that as temperature decreases, the L_α_ phase first transforms to a conventional tilted L_β_ phase and then further changes to form a new folded configuration. Although the transition temperature does not strictly agree with experimental results, the two transitions are consistent in the computational and experimental results.

The distribution of water and glycerol molecules is also crucial for understanding the mechanism. As shown in Figure 5a,d and Figure S8 (Supporting Information), both water and glycerol molecules only arranged the outside of solvo‐phobic region from −20 to 90 °C. In contrast, at 120 °C, a small amount of water and glycerol penetrated the solvo‐phobic area. Therefore, it is clear that glycerol does not affect the aggregation of the SDA alkyl chain at low temperatures, and that the mixed water and glycerol molecules provide a perfect anti‐freezing medium for cryogenic self‐assembly.

Figure 5e shows the radial distribution function (RDF) between the hydroxyl of SDA and the water. The two peaks correspond to the nearest water molecule solvent layers of the two hydroxyl groups of SDA. The RDF between the hydroxyl of SDA and glycerol is depicted in Figure 5f. The position of the peaks shows that glycerol and water formed a consistent solvent layer and that no additional solvent layer was formed. The values of the peaks reveal that the first solvent layer was dominated by water. Thus, it is concluded that the SDA hydrophilic group mainly interacts with water, rather than glycerol.

To further illustrate the interaction of SDA with water and glycerol, we calculated the number of H‐bonds formed by SDA, as shown in Figure 5g. The number of H‐bonds between SDA and water was around 2, while that between SDA and glycerol was only ≈0.5, demonstrating that SDA mainly forms H‐bonds with water, rather than glycerol, though they have the same volume ratio. As the temperature increased, the number of H‐bonds between SDA and water started to decrease at 20 °C and stabilized at around 1.6 at 60 °C, while that between SDA and glycerol slightly increased at 40 °C.

When the glycerol content increased to 70 and 90 vol%, the width of SDA density distribution slightly increased (Figure 5h; Figure S9, Supporting Information), which is in line with the experimental result of the increase in bilayer thickness. The density distribution of glycerol and water (Figure 5I; Figure S10, Supporting Information) shows that glycerol still did not penetrate the solvo‐phobic area. With the increase in glycerol content, the number of H‐bonds between SDA and glycerol linearly increased, while that between SDA and water decreased (Figure 5j). Note that the total number of H‐bonds decreased from 2.6 to 1.7 when glycerol content increased from 50 to 90 vol%. The reduced number of H‐bonds weakened the anchoring force of SDA, which could be the reason that the bilayer thickness increased with glycerol content. The critical micelle concentration (cmc), determined from the plot of surface tension against SDA concentration (Figure S11, Supporting Information), exhibited an increase from 3.5×10^‒2^ to 4.9×10^‒2^ mM as the glycerol content rose from 50 to 70 vol%. This rise in cmc with increasing glycerol content implies a reduction in the self‐assembly capability of the cosolvent, which is in line with the observed changes in H‐bonds.

**Scheme 2:**
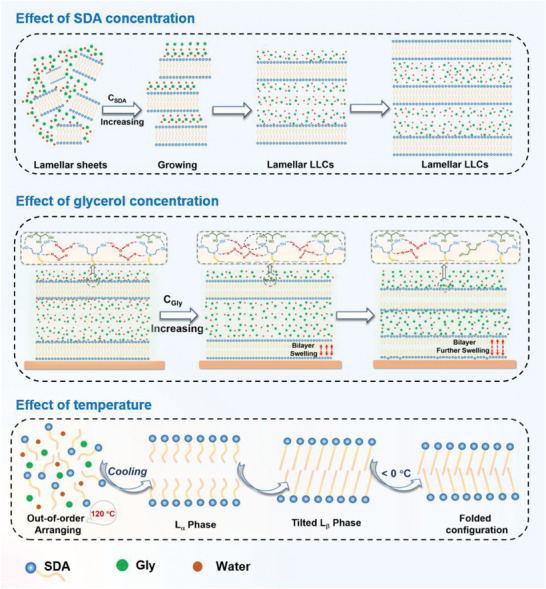
Schematic illustration of SDA self‐assembly behavior in water/glycerol binary solvent.

From our experimental and simulation results, we know that both the ultra‐long alkyl chain and head group of SDA play a crucial role in the formation of lamellar nanostructures, as illustrated in **Scheme** **2**. The ultra‐long alkyl tail showed a strong solvo‐phobic effect, thus imparting its excellent aggregation ability in the mixture of water and glycerol. Moreover, the head group of SDA could form multiple H‐bonds with solvent molecules, which not only guaranteed good solubility but also well stabilized the whole SDA molecule to maintain the aggregation. As such, SDA showed a superior ability to form lamellar nanostructures, which generated lamellar sheets at only 0.1 wt%. As the SDA concentration increased, more lamellar sheets were formed and stacked in a long‐range order generating LLCs. The bilayer thickness did not change, but the solvent thickness decreased with SDA concentration owing to the squeezing of lamellar layers. The SDA head group was prone to forming H‐bonds with water, rather than glycerol. Thus, as the glycerol content increased, the total number of H‐bonds decreased, which weakened the stabilization of the whole molecule and resulted in the swelling of the lipid bilayer. At the extremely high temperature of 120 °C, SDA molecules were arranged out of order in a solvo‐phobic region, and both water and glycerol penetrated the solvo‐phobic region. As temperature decreased below zero, LLCs with the liquid‐crystalline L_α_ phase, tilted L_β_ phase, and a new folded configuration of the alkyl chain were obtained consecutively. The two peaks observed in WAXS at subzero temperatures demonstrate the co‐existence of the tilted L_β_ phase and the folded configuration, which could ascribe to the slow transition speed at low temperatures.

### 2.5 Rheological Properties

**Figure 6:**
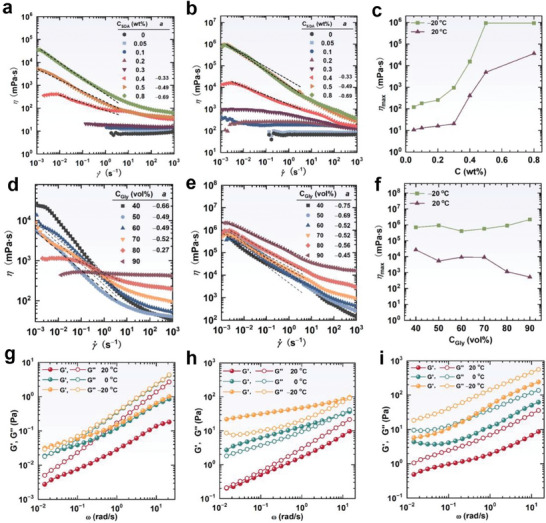
Rheological properties of SDA fluids. Flow curves of SDA fluids at different concentrations with 50 vol% glycerol at a) 20 °C, b) −20 °C, and c) the maximum viscosity as a function of SDA concentration. Flow curves of 0.5 wt% SDA fluids with different glycerol contents at d) 20 °C, e) −20 °C, and f) the maximum viscosity as a function of glycerol content. Dynamic rheology of g) 0.5 wt% and h) 1.0 wt% SDA solution with 50 vol% glycerol, and i) 0.5 wt% SDA solution with 70 vol% glycerol.

**Figure 7:**
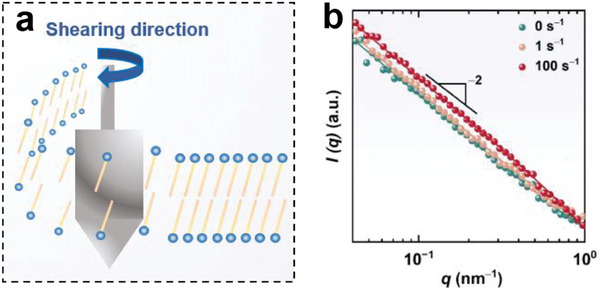
a) Schematic illustration of the structure change of lamellar lyotropic liquid crystals upon shearing. b) Rheo‐SANS profiles of 0.5 wt% SDA solution with 50 vol% glycerol at various shear rates at −20 °C.

The formation of lamellar nanostructures imparts unique rheological properties to the bulk solution. **Figure** **6**a shows the flow curves of various fluids containing 50 vol% glycerol but different SDA concentrations at 20 °C. Fluids with SDA concentrations less than 0.4 wt% behaved as a Newtonian fluid with low viscosity. When the SDA concentration reached 0.4 wt%, a viscosity plateau followed by an obvious shear‐thinning was observed, showing the characteristics of a non‐Newtonian fluid. This result may be due to the formation of LLCs. The viscosity decrease with increasing shear rate can be attributed to the shear‐induced orientation, i.e., lamellae domains aligned and stretched in flow direction (**Figure** **7**a).^[^ ^21^ ^]^

Figure 6b shows the corresponding rheology curves at −20 °C. The maximum viscosity (η_max_) was obtained and plotted with the SDA concentration (Figure 6c). Viscosity increased with the temperature decrease, which can be ascribed to the configuration transition of SDA alkyl chains and the improved stacking order of lamellar layers at low temperatures. Shear‐thinning behavior is significantly important for applications. To examine this behavior, we fitted flow curves between the shear rate where viscosity obviously decreased and 3 s^−1^ according to the equation η  =  *k*γ^−*a*^, where the exponent reflects the shear‐thinning property.^[^ ^22^ ^]^ The exponent value rose with the SDA concentration increase and/or temperature reduction (Figures 6a,b), potentially owing to the better stacking order of lamellar layers, resulting in easier shear orientation. In addition, Rheo‐SANS results show that the lamellar structure remained stable as shear rate changed in a large range from 0 to 100 s^−1^ for various samples at 20 or −20 °C (Figure 7b; Figure S12a,b, Supporting Information), suggesting they have excellent shear‐resistance.

Figures 6d−f show the solvent effect on the flow curves. The shear‐thinning behavior decayed with the glycerol content increase. The 0.5 wt% SDA solution with 90 vol% glycerol was completely transformed into a Newtonian fluid without shear‐thinning behavior at 20 °C. However, when the temperature decreased to −20 °C, the viscosity increased, and the shear‐thinning behavior recovered. Namely, the exponent value decreased with glycerol content rise but increased with a temperature reduction. This is also related to the microstructural variation, where a lower glycerol content and temperature enhanced the lamellar layer stacking order.

Dynamic rheological properties were investigated by oscillating shear measurements (Figures 6g−i). The 0.5 wt% SDA solution with 50 vol% glycerol was a viscous fluid at 20 °C because the storage modulus (*G*′) was smaller than the loss modulus (*G*′′) within the range of accessible frequencies. As the temperature decreased to 0 and −20 °C, both the values of *G*′and *G*′′ increased and a crossover appeared, which resulted from the strengthening of the microstructures at lower temperatures. When the SDA concentration increased to 1.0 wt%, the solution behaved as a cryo‐gel at −20 °C with *G*′ > *G*′′. Further increases in the SDA concentrations (Figure S13a,b, Supporting Information) led to the improvement of rheological properties. Nevertheless, the increase in glycerol content weakened the rheological properties. For example, the 0.5 wt% SDA solution with 70 vol% (Figure 6i) or 90 vol% glycerol (Figure S13c, Supporting Information) only showed the characteristic of viscous fluids at temperatures ranging from 20 to −20 °C.

SDA lamellar nanostructure has powerful thickening ability, and its rheological properties are determined by the microstructures that can be easily regulated by SDA concentration, glycerol content, and temperature. Upon decreasing the temperature to subzero values, rheological properties are dramatically improved due to the formation of folded configuration of SDA alkyl chains and the enhancement of lamellar layer stacking regularity. These lamellar nanostructures can produce a series of cryogenic viscous, viscoelastic, and gel‐like fluids, and can also be exploited to produce other new materials, i.e., use to mimic cell membranes or use as templates to prepare nano‐materials in extremely cold environments.

## 3 Conclusion

Extending self‐assembly to subzero temperatures is significant to enrich the understanding of supramolecular self‐assembly under extreme environments, and to create novel materials. We have reported the self‐assembly behavior and rheological properties of the specifically tailored surfactant SDA in water/glycerol binary solvent down to subzero temperature of −70 °C. SDA formed lamellar sheets at 0.1 wt% and lamellar liquid crystals at 0.5 wt%, two orders of magnitude lower than conventional systems. Glycerol molecules could not penetrate the solvo‐phobic area, which mixed with water molecules arranging outside of the solvo‐phobic area, providing an ideal anti‐freezing environment for self‐assembly. The SDA head group was prone to forming H‐bonds with water, rather than glycerol. Consequently, the overall quantity of H‐bonds decreased with glycerol content increase, thereby weakening the stabilization of SDA molecules and resulting in an expansion of the lipid bilayer. When the temperature falls below 0 °C, LLCs with the L_α_ phase, tilted L_β_ phase, and a new folded configuration were obtained consecutively. The microstructure of LLCs confers unique rheological properties on the bulk fluid. As such, a series of cryogenic materials, including viscous, viscoelastic, and gel‐like fluids, were created; this has potential applications for use in extremely cold environments.

The authors have cited additional references within the Supporting Information.^[^ ^23^, ^24^, ^25^, ^26^ ^]^

## Conflict of Interest

The authors declare no conflict of interest.

## Supporting information

Supporting Information

Supplementary Movie 1

Supplementary Movie 2

Supplementary Movie 3

## Data Availability

The data that support the findings of this study are available from the corresponding author upon reasonable request.
