# Supplementary material for: Self‐Assembling Anti‐Freezing Lamellar Nanostructures in Subzero Temperatures
Source: Adv Sci (Weinh). 2024 Feb 17;11(17):2309020. doi: 10.1002/advs.202309020 (PMC11077679; doi:10.1002/advs.202309020)
Supplement: Supplementary file 1 — Supporting Information [file ADVS-11-2309020-s002.pdf]

ADVANCED SCIENCE
Open Access

Supporting Information

for *Adv. Sci.*, DOI 10.1002/advs.202309020

Self-Assembling Anti-Freezing Lamellar Nanostructures in Subzero Temperatures

Hongyao Yin, Weiluo Guo, Runxi Wang, James Doutch, Peixun Li, Qiang Tian, Zhuo Zheng, Lingzhi Xie and Yujun Feng**

Supporting Information
©Wiley-VCH 2021
69451 Weinheim, Germany

Self-Assembling Anti-Freezing Lamellar Nanostructures in Subzero Temperatures

Hongyao Yin*, Weiluo Guo, Runxi Wang, James Doutch, Peixun Li, Qiang Tian, Zhuo Zheng, Lingzhi Xie, and Yujun Feng*

Abstract: The requirement for cryogenic supramolecular self-assembly of amphiphiles in subzero environments is a challenging topic. Here, the self-assembly of lamellar lyotropic liquid crystals (LLCs) are presented to a subzero temperature of −70°C. These lamellar nanostructures are assembled from specifically tailored ultra-long-chain surfactant stearyl diethanolamine (SDA) in water/glycerol binary solvent. As the temperature falls below zero, LLCs with a liquid-crystalline L_α phase, a tilted L_β phase, and a new folded configuration are obtained consecutively. A comprehensive experimental and computational study was performed to uncover the precise microstructure and formation mechanism. Both the ultra-long alkyl chain and head group of SDA play a crucial role in the formation of lamellar nanostructures. SDA head group is prone to forming hydrogen bonds with water, rather than glycerol. Glycerol cannot penetrate the lipid layer, which mixes with water arranging outside of the lipid bilayer, providing an ideal anti-freezing environment for SDA self-assembly. Based on these nanostructures and the ultra-low freezing point of the system, a series of novel cryogenic materials were created with potential applications in extremely cold environments. These findings would contribute to enriching the theory and research methodology of supramolecular self-assembly in extreme conditions and to developing novel anti-freezing materials.

DOI: 10.1002/advs.202309020

Table of Contents

1. Materials, characterizations and methods
2. Schematic diagram of molecular dynamics for bilayer formation
3. Appearance of SDA solution with various glycerol content
4. Fluidity of SDA solutions at various subzero temperatures
5. POM images of SDA solutions
6. SANS profiles of SDA solutions at various temperatures
7. Supplementary molecular dynamics simulation results
8. Surface tension profiles of SDA solutions
9. Rheo-SANS profiles of SDA solutions at different shear rates
10. Dynamic rheology of SDA solutions at various temperatures

1. Materials, characterizations and methods

Materials. stearyl diethanolamine (SDA, ≥ 98%) were purchased from Shanghai Adamas Reagent Co., Ltd. (Shanghai, China). Glycerol (Gly, ≥ 99%) were purchased from Tianjing BODI Chemical Engineering Co., Ltd. (Tianjing, China). Deuterium oxide (99.9 atom% D) was purchased from Sigma-Aldrich. All chemicals were used without further treatment. The water used in this work has a resistance of 18.25 MΩ·cm, which was double-deionized using an ultrapure water purification system (CDUPT−III, Chengdu Ultrapure Technology Co., Ltd., China).

Preparation of fluids. A designated amount of SDA was added to a water/glycerol binary solvent followed by stirring at room temperature until SDA complete dissolving. The fluids were then stored at room temperature for another 24 h prior to other measurements.

Determination of SDA solubility in water/glycerol binary solvent. 0.5 wt% SDA solutions were prepared in a series of water/glycerol binary solvents with various glycerol content. The transmittance of the solutions was recorded on a UV−6100 double beam spectrophotometer (Shanghai Mapada Instruments Co. Ltd., China) at a fixed wavelength of 550 nm. The temperature of the measured solution was controlled by an external Julabo circulating bath at 20°C. The solubility was evaluated by the transmittance.

Determination of freezing temperature. Freezing temperature was determined according to engine coolant freezing temperature determination method (SH/T0090-91) of China on a XY-117 freezing point, turbidity point and crystal point detector (Dalian Xinyi Automation Instrument Co., Ltd., China). Each sample was measured twice, and the average value was taken as the final freezing point when the difference between each measured value and the average value is no more than 0.3°C.

Fluidity at subzero temperatures. SDA fluid was dyed blue using methylene blue, which was transferred to an injection syringe and cooled down to −150°C and held for 2 min using liquid nitrogen bath. The real temperature of the fluid was tested by a temperature monitor inserted into the fluid (Shandong Renke Measurement and Control Technology Co., Ltd, Jinan, China). Then the syringe was taken out, whose temperature was naturally recovered. When the temperature reached the set value, a pressure was applied on the fluid to check its fluidity.

Small-angle neutron scattering measurement. Small-angle neutron scattering (SANS) measurements were conducted on the ZOOM instrument at the ISIS pulsed neutron source (ISIS, RutherfordAppleton Laboratory, STFC, Didcot, Oxfordshire, UK). The neutron scattering of the samples were collected on a two-dimensional detector placed at 4 m from the sample giving scattering vector q range from 0.009 to 0.29 $Å^{-1}$, using the time-of-flight technique with neutrons of wavelength 1.75−16.5 Å. The samples were prepared in deuterium oxide/glycerol to optimize the contrast with SDA. The samples were placed in clean disc-shaped quartz cells (Hellma) with 2 mm path length for the measurements without shearing, whereas they were loaded in a Couette quartz cell in an Anton Paar MCR 501 rheometer for the measurements with shearing. Raw data were corrected for detector efficiency, transmission, scattering from the empty cell, and corresponding background using Mantid data reduction software. Then the data were converted to the differential scattering cross-sections by calibration of the beamline using a polymer standard of well-known cross section. Data analysis of the SANS profiles was performed with the SasView software (https://www.sasview.org/).

Small-angle X-ray scattering measurements. Small-angle X-ray scattering (SAXS) measurements were performed on a SAXSpace instrument (Anton Paar, Austria, Cu−*K*α, λ = 0.154 nm), equipped with a Kratky block-collimation system and a Mythen2 R 1K detector. Samples were filled into a 1 mm diameter quartz capillary before measurements. The scattering curves of solvents in the same capillary were recorded as background. The data were normalized to the incident primary beam intensity and correct for background scattering, and were desmeared using the SAXSquant software developed by Anton Parr.

Wide-angle X-ray scattering measurements. The wide-angle X-ray scattering (WAXS) measurements were performed on a synchronous diffraction laboratory instrument, Nano-inXider (Xenocs, France), equipped with a micro-focus source generating X-rays of a wavelength λ = 1.542 Å (Genix3D), operating at 50 kV and 0.6 mA. Each sample was exposed to the beam for 600 s using the Nano-inXider and 1 s at the synchrotron. All solution samples were transferred to the borosilicate capillaries (1.5 mm outer diameter, 0.01 mm wall thickness) at a sample-to-detector distance of 79 mm. Statistical analysis was performed using FIT2D.

Cryogenic scanning electron microscopy observation. Cryogenic scanning electron microscopy (cryo-SEM) experiments were performed on a FEI Quanta 450 scanning microscope (FEI Company, US) equipped with a Quorum cryo-stage PP3000T. The samples for observation were prepared by a freeze-drying method: samples were first frozen in liquid nitrogen (−185°C) for 30 s, and then were transferred to a chamber to sublimate the moisture under vacuum at −90°C for 10 min. Subsequently, the samples were coated with Au and then observed at −140°C at an acceleration voltage of 5 kV.

Polarizing optical microscopy visualization. Crystalline morphologies were determined on a Nikon LV100NPOL polarizing optical microscope (Nikon Corporation, Japan) at room temperature, and samples were visualized in a polarized light rotated two degrees. All the samples were prepared in a slide groove with high transmittance, keeping stable solution surface and phase states. All the polarizing microscope graphs were viewed at 20× magnification under a steady cast of light.

Differential scanning calorimetry measurement. Differential scanning calorimetry (DSC) measurements were performed with a differential scanning calorimeter Q2000 (TA Instruments, US). Nitrogen at the flow rate of 50 mL·min^{-1} was used as a purge gas. Approximately 10 mg of sample was taken to measure. Samples were first heated from room temperature to 80°C at the heating rate of 10 °C·min^{-1}, then were cooled to −70°C at the cooling rate of 10 °C·min^{-1} and remained at this temperature for 10 min. Afterwards, these samples were heated to 80°C again at the rate of 2 °C·min^{-1}.

Rheological test. Rheograms were recorded on a Physica MCR 302 (Anton Paar, Austria) rotational rheometer equipped with CC27 concentric cylinder geometry. All samples were equilibrated at set temperatures for 10 min prior to test. The steady shear viscosity data were continuously collected in the shear rate ranging from 10^{-3} to 10^{3} s^{-1} with a ramp rate following the logarithm law, and the oscillatory-shear experiments were performed in the linear viscoelastic regime that was previously determined from dynamic stress sweep tests. All measurements were carried out in the stress-controlled mode, and standard oil was used to calibrate the instrument before measurements.

Molecular dynamics simulation. The schematic of the molecular dynamics (MD) simulation system used in this work is shown in **Figure S1**. It is a system for the formation of an SDA bilayer in mixed solvents of glycerol and water. We used the OPLS all-atom force field[23] to model the interaction of SDA and glycerol, which has been demonstrated by numerous simulations to be suitable for modelling water and organic systems. We also used the Tip3p water model[24] that matches it. The interactions between different atoms are obtained by the Lorentz-Berthelot mixing rule.[25] All simulations were performed using Gromacs software.[26] We varied the temperature (−20°C to 120°C) and the ratio of glycerol to water (5:5, 7:3 and 9:1) to investigate their effects on SDA self-assembly separately. Each simulation was first run for 100−200 ns to reach equilibrium (depending on the temperature) and subsequently run for 200 ns for analysis.

Figure S1 shows a schematic of the formation of SDA bilayer structure in different ratios of water and glycerol solvents. The middle of the model is a bilayer structure with hydrophilic groups facing outwards and hydrophobic carbon chains facing inwards. There are 100 SDA molecules in the model, 50 on each side. On both sides there is a mixture of glycerol and water, with 50%, 70% and 90% glycerol by volume. The dimensions of the simulated system are $3.65 \times 3.65 \times 17.5$ nm^3 in each of the three dimensions. Periodic boundary conditions are applied in all three dimensions, and the size of the model is large enough to ignore the effects from periodicity. During the simulations, a Nose-Hoover thermostat is used to maintain the system temperature at the same level as the experiment (−20, 0, 20, 40, 60, 90, 120°C). The volume ratio of glycerol is achieved by reducing the number of molecules of water. The Leap-Frog algorithm is applied with 2 fs as time step to integrate Newton's equations of motion. The electrostatic interactions in inverse space are calculated using a 0.1 nm fast fourier transform grid. All simulations are calculated from the initial structure. The NPT ensemble is first run for 100−200 ns at 1 atmosphere to equilibrate the system and form the SDA self-assembled structure. Subsequently, the NVT ensemble is used to fix the size of the simulation system and run 200 ns for statistical analysis

2. Schematic diagram of molecular dynamics for bilayer formation

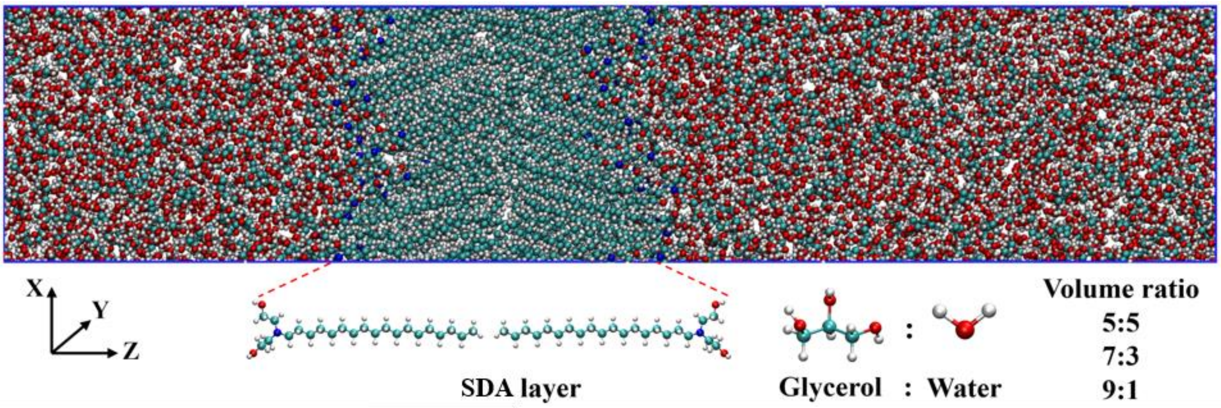

Figure S1. The schematic diagram of molecular dynamics simulations for SDA bilayer formation in water/glycerol binary solvent.

3. Appearance of SDA solution with various glycerol content

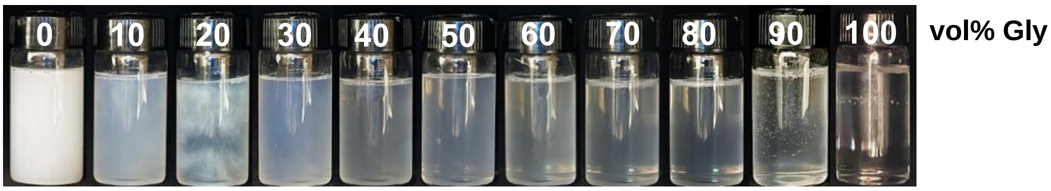

vol% Gly

Figure S2. Snapshots of the appearance of 0.5 wt% SDA solutions with various glycerol content at 20°C.

4. Fluidity of SDA solutions at various subzero temperatures

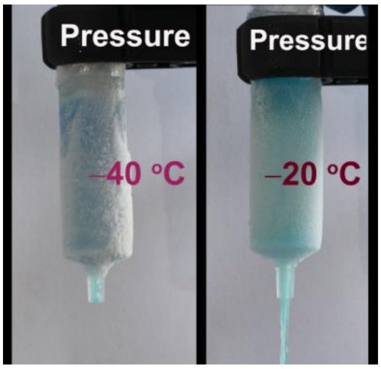

Figure S3. Fluidity of 1.0 wt% SDA solution with 50 vol% glycerol at −40°C and −20°C. The results show that this solution cannot flow at −40°C but at −20°C.

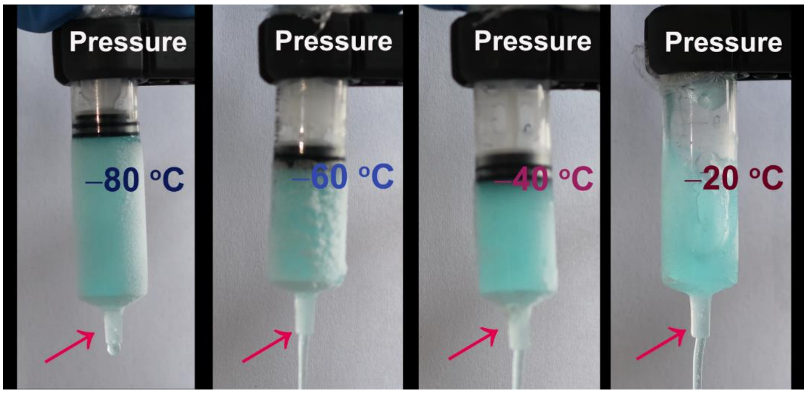

Figure S4. Fluidity of 1.0 wt% SDA solution with 90 vol% glycerol at −80°C, −60°C, −40°C and −20°C. The results show that this solution cannot flow at −80°C but at −60°C or above.

5. POM images of SDA solutions

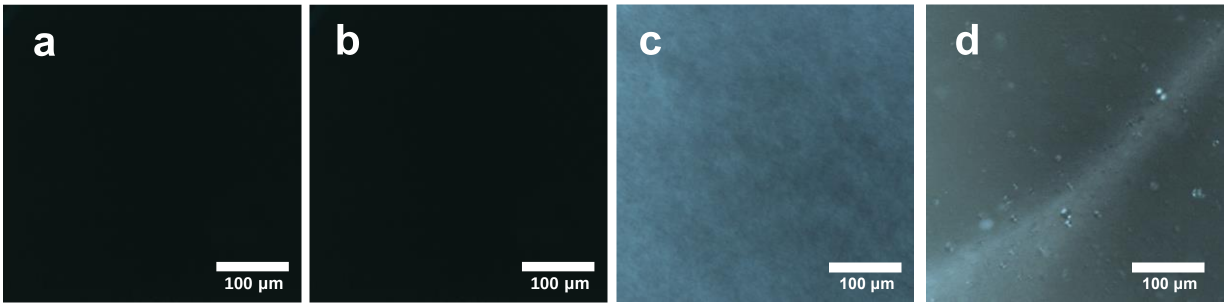

Figure S5. Polarized optical micrograph of (a) 0.1 wt% and (b) 0.3 wt% SDA solutions with 50 vol% glycerol, and (c) 0.5 wt% SDA solution with 70 vol% glycerol, and (d) 0.8 wt% SDA solution with 90 vol% glycerol at 20°C.

6. SANS profiles of SDA solutions at various temperatures

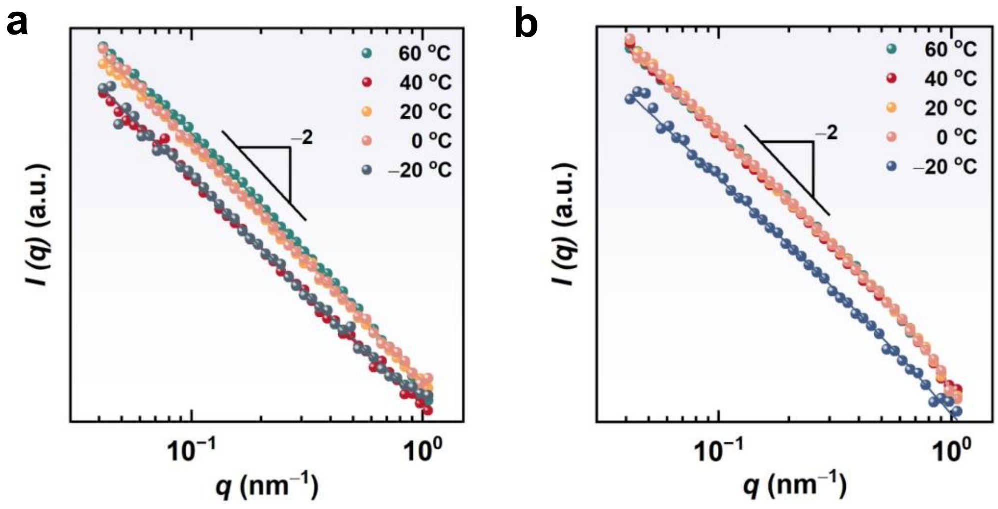

Figure S6. SANS profiles of (a) 0.5 wt% and (b) 0.8 wt% SDA solutions with 50 vol% glycerol at various temperatures.

7. Supplementary molecular dynamics simulation results

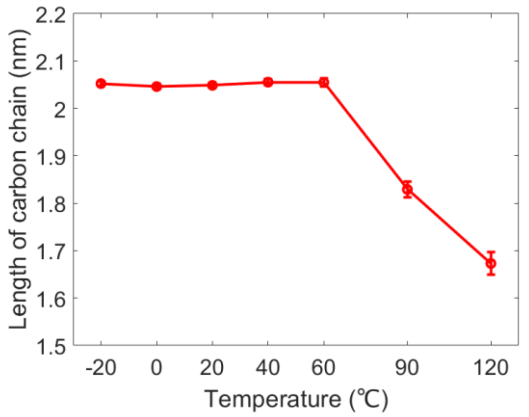

Figure S7. Variation of SDA carbon chain length with temperature in water/glycerol binary solvent with 50 vol% glycerol.

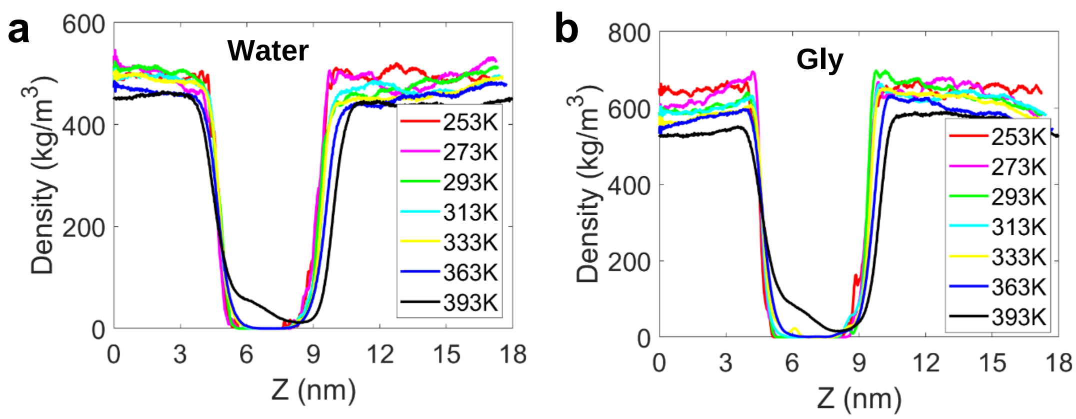

Figure S8. Density distribution of (a) water and (b) glycerol at various temperatures for SDA solution with 50 vol% glycerol.

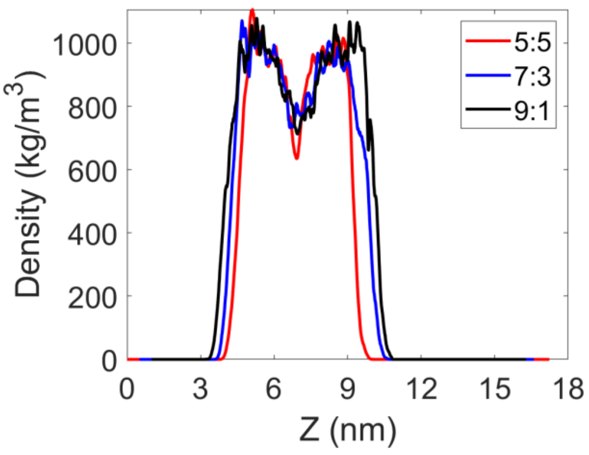

Figure S9. Density distribution of SDA solutions with different glycerol to water ratios at 20°C.

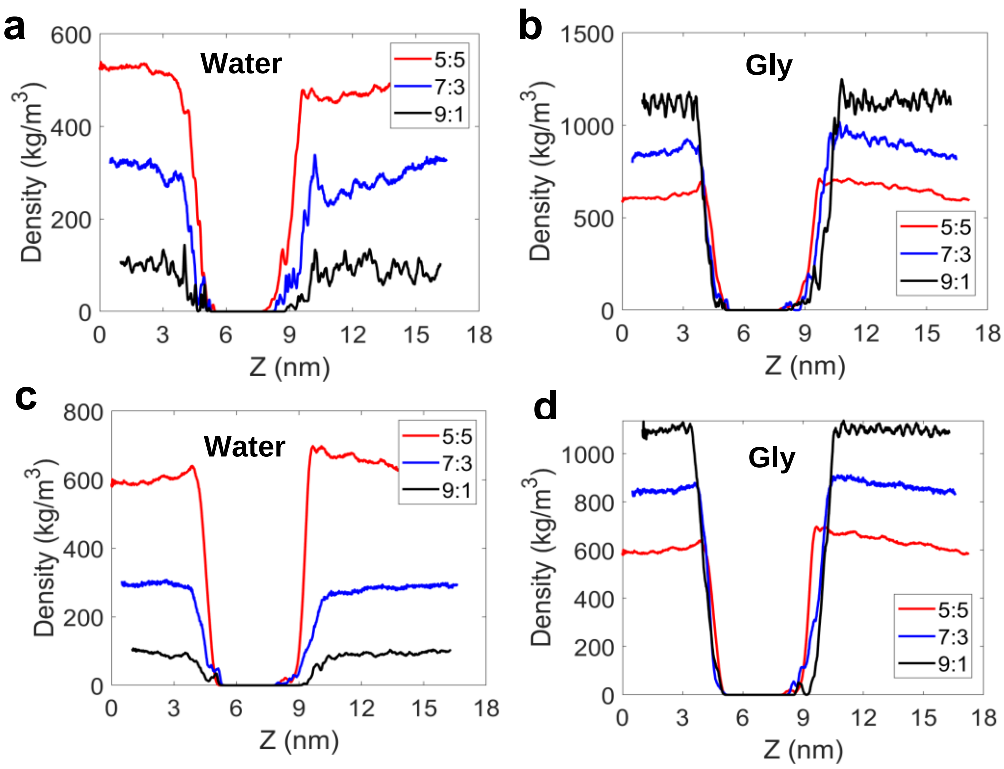

Figure S10. Density distribution of (a) water and (b) glycerol for SDA solutions with different glycerol to water ratios at −20°C. Density distribution of (c) water and (d) glycerol for SDA solutions with different glycerol to water ratios at 20°C.

8. Surface tension profiles of SDA solutions

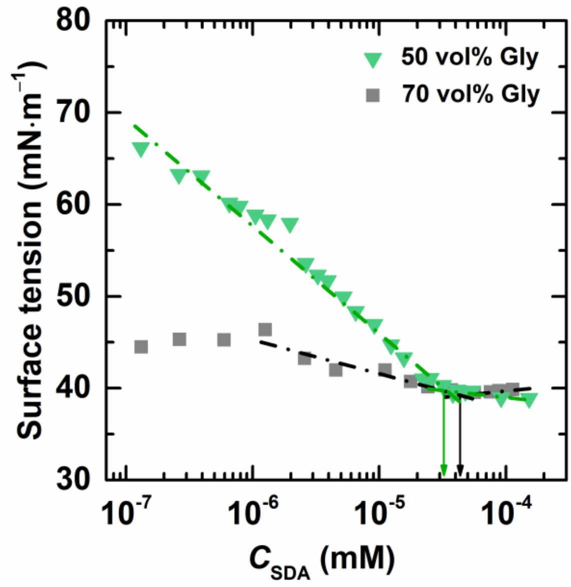

Figure S11. Surface tension as a function of SDA concentration in water/glycerol binary solvents with 50 vol% and 70 vol% glycerol at 20 ºC.

9. Rheo-SANS profiles of SDA solutions at different shear rates

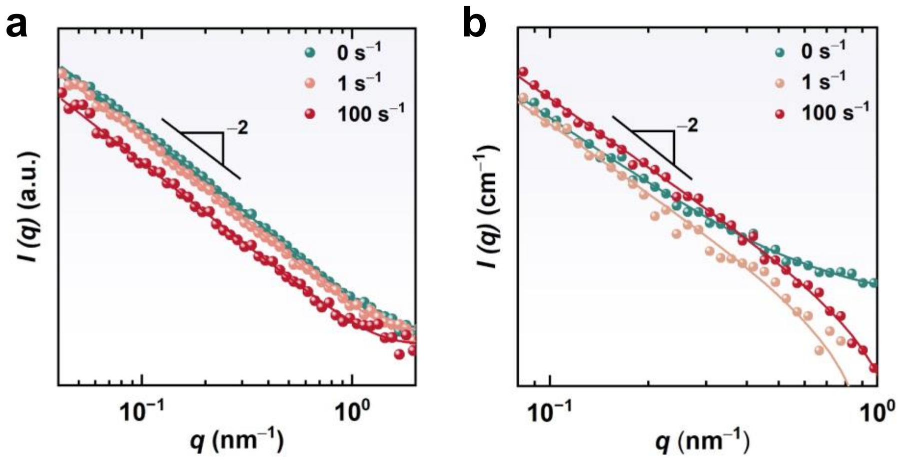

Figure S12. Rheo-SANS profiles of 0.5 wt% SDA solutions with (a) 50 vol% glycerol at 20°C, and (b) 70 vol% glycerol at −20°C at different shear rates.

10. Dynamic rheology of SDA solutions at various temperatures

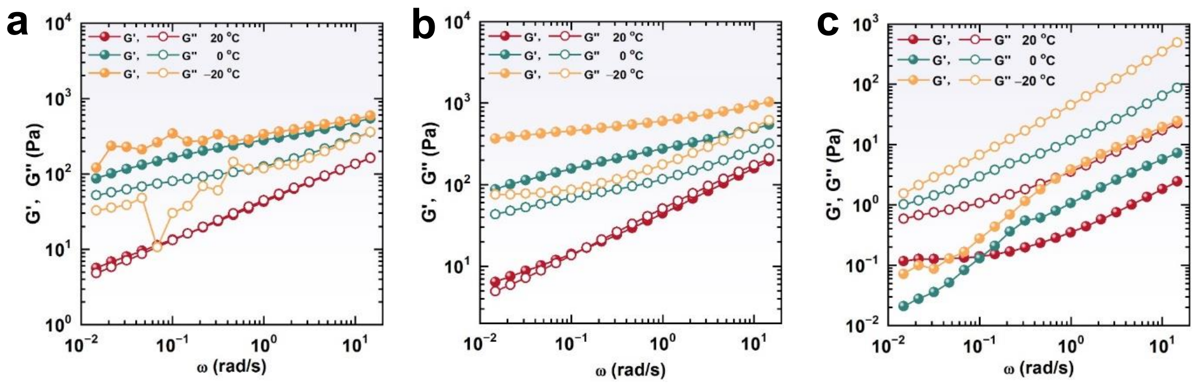

Figure S13. Dynamic rheology of (a) 2.0 wt% and (b) 3.0 wt% SDA solutions with 50 vol% glycerol, and (c) 0.5 wt% SDA solution with 90 vol% glycerol.

References

[23] W. L. Jorgensen, J. Tirado-Rives, *Proc. Natl. Acad. Sci.* **2005**, *102*, 6665–6670.
[24] P. Mark, L. Nilsson, *J. Phys. Chem. A* **2001**, *105*, 9954–9960.
[25] H. A. Lorentz, *Ann. Phys. Berlin*, **1881**, *248*, 127–136.
[26] B. Hess, C. Kutzner, D. van der Spoel, E. Lindahl, *J. Chem. Theory Comput.* **2008**, *4*, 435–447.

Author Contributions

Hongyao Yin: conceptualization, validation, funding acquisition, project administration, writing of original draft
Weiluo Guo: investigation, data curation, formal analysis
Runxi Wang: investigation, data curation
James Doutch: investigation, methodology, validation
Peixun Li: methodology, resources
Qiang Tian: investigation, methodology
Zhuo Zheng: methodology, resources
Lingzhi Xie: methodology, resources
Yujun Feng: funding acquisition, project administration
